# Schooling for Pupils with Autism Spectrum Disorder: Parents’ Perspectives

**DOI:** 10.1007/s10803-020-04496-2

**Published:** 2020-04-10

**Authors:** Lotta Anderson

**Affiliations:** Department of School Development and Leadership, Faculty of Education and Society, Malmö University, Nordenskiöldsgatan 10, 205 06 Malmö, Sweden

**Keywords:** Autism spectrum disorder, Gender, Grades, Educational support, Parents perspectives, School absence

## Abstract

The current study, based on a survey of 1799 parents, explored parental perspectives of school absence in relation to approved grades, challenges, demands and obstacles in education for children with autism spectrum disorder. The results revealed a relatively high rate of school absenteeism for reasons other than illness. Girls had higher rates of absenteeism than boys for short durations of absence. Absenteeism was primarily caused by a lack of teacher competence regarding autism and inadequate adaptation of teaching. There were no significant differences between genders in approved grades, but the rate of failure to achieve approved grades was approximately 50%. The most common form of educational support was support from special needs teachers and adapted pedagogy.

## Introduction

The current study sought to contribute to knowledge about pupils with autism spectrum disorder (ASD) and their schooling in Sweden by examining parental perspectives. Schooling situations for pupils with ASD have been examined in research settings, as well as by school authorities and media. In order for schooling to be effective, it is widely assumed that certain educational, social and physical conditions must be met (Holmqvist 2009; Göransson and Nilholm 2014).

The Swedish National Agency for Education does not collect statistics about pupils with disabilities; therefore, there is no overall picture of the number of pupils with ASD in Swedish schools. In 2019, 115,000 children were born in Sweden. Bejnö et al. (2019) indicate that the number of children diagnosed with ASD in developing countries over the last two decades has increased and the number of children can be expected to reach 1–3% of the population.

In primary school grades 1–9, 1,068,274 pupils receive their education. Of the 112,731 pupils in grade 9, 75% meet the knowledge requirements in all subjects. Girls reach the knowledge requirements to a somewhat higher degree (78.3%) than boys do (72.8%). Of pupils who have completed secondary school, 104,603 pupils, (the proportion of girls constitutes 47%), 73, 4% of the pupils have basic eligibility for higher studies (National Agency for Education 2018, 2019).

In the primary school for learning disabilities, 11,140 pupils receive their education, of which 38% are girls. In the secondary school for learning disabilities, 6140 pupils are educated, of which 40% are girls. Pupils in primary school for learning disabilities who read subjects receive grades if the pupils or their parents request it. In the secondary school for pupils with disabilities, pupils receive grades on each completed course. There are no overall statistics of pupils’ grades in special schools for learning disabilities (National Agency for Education 2018, 2019). Swedish School Inspectorate notes in a report (2016) that pupils with extensive school absenteeism amount to 20,000 pupils. However, school absenteeism has increased in recent years.

In Sweden, pupils with ASD have traditionally been placed in special schools for pupils with intellectual disability (ID) or have been subject to special solutions due to their reduced ability to understand the social context of schools compared with other pupils, and distress caused by excessive sensory stimulation. More recently, several directives and policies in Sweden (Education Act, SOU 2010: 800) have specified that only pupils with diagnosed cognitive disabilities can be placed in special schools for pupils with ID. This has resulted in a substantial number of pupils with ASD with typical cognitive ability being expected to complete schooling at typical primary and secondary schools under the same conditions as pupils without ASD. This puts substantial pressure on pupils with ASD, and the situation has been reported to impose several difficulties. The Autism and Asperger Association (2013) in Sweden periodically administers school questionnaires, and the results have confirmed this phenomenon. The results of these questionnaires indicated that only 35% of parents of children with ASD in mainstream schools believe this type of school is best for their children. A large proportion of children with ASD exhibited absenteeism (33%) and the rate of goal fulfillment was low in various subjects, including Swedish, English and mathematics (44%) (The Autism and Asperger Association (2013). However, in contrast to the views expressed by parents, the European Agency (2018) reported that “Learners with disabilities educated in inclusive settings may perform academically and socially better than learners educated in segregated settings” (p. 63). Linton (2015) reported that there is no research in Sweden regarding the educational situation for pupils with autism. It is therefore difficult to draw conclusions about successful educational solutions for pupils with ASD. Linton (2015) emphasizes that there is a gap between the intention to include pupils with ASD in mainstream classrooms and how these intentions are implemented. For example, Linton (2015) argues that there is a lack of adequate support for pupils, in accord with a report from The Autism and Asperger Association (2013).

The Swedish Schools Inspectorate (2012) reported deficiencies in providing adequate support to enable pupils with ASD to reach proficiency. It has been found that teaching was not adequately based on pupils’ abilities and needs, and pupils found it difficult to achieve goals (National Board of Health 2010; Petersson 2015). These results are in contrast with policy intentions highlighting inclusion of all students as a goal. The APA’s DMS-5 diagnostic criteria (McPartland et al. 2012) state that ASD involves abnormal functioning of the cerebral network, which explains the lack of integration of perception, motor function and cognition, which leads to different behavior and mindset. This has been confirmed by a number of other researchers, including Fernell et al. (2010) and Andersson Westman, Gillberg and Miniscalco (2013).

## Previous Research on Pupils with ASD

Previous research has suggested opportunities to develop education for pupils with autism and developmental disabilities (Holmqvist 2009), but no previous studies have identified problems with the school situation while also suggesting a foundation for improving it. Previous research can be divided into three main areas: mainstream school and special solutions for pupils with ASD, school absence for pupils with ASD, and challenges, demands and obstacles in education.

### Mainstream School and Special Solutions for Pupils with ASD

There is currently widespread dissatisfaction with segregated solutions in schools (i.e., different school systems for pupils who are considered to be “typically developing” and those considered to exhibit “abnormal development”) among both professionals and parents (Linton 2015; European Agency 2018; The Autism and Asperger Association 2013). Göransson and Nilholm (2014) argued that research and experience indicate that the ways in which classes and teachers function are more important for pupils than the ways in which schools are organized at a general level. In addition, the researchers suggested that avoiding special solutions outside typical classrooms could be one aspect of inclusion (Göransson and Nilholm 2014). The definition of inclusion often has broader implications for the school beyond the placement of learners with disabilities in mainstream classrooms. Inclusion also means that every pupil must have access to a good school situation and the school must meet the social and academic needs of learners with disabilities as well as creating inclusive communities in which inequality is not an obstacle.

Both the Convention on Rights of the Child (United Nations 1989) and the Convention on the Rights of Persons with Disabilities (United Nations 2006) clearly outline the right of children with disabilities to inclusive education. In addition, Article 24 (United Nations 2006) specifies the right to inclusive education. Moreover, the European Agency (2018) suggested that pupils with disabilities educated in inclusive settings might perform better, academically and socially, than pupils educated in segregated settings. However, Waddington and Reed’s (2017) findings indicated that typically developing pupils do not achieve greater academic success than children do in special solutions settings. The European Agency (2018) suggested that prerequisites for leisure activities, starting higher education, obtaining employment and becoming financially independent after completion of education increase after education in inclusive settings. Grogan (2015) pointed out that if pupils with ASD are not qualified for higher education, tertiary institutions may face difficulties meeting the unique needs of these pupils. Specifically, transition, academic achievement and social skills can create obstacles for pupils with ASD.

Teixeira De Matos and Morgado (2016) reported that inclusion in mainstream schools is influenced by a positive attitude towards pupils with ASD and their behavior, their attendance in the school or class, and their overall acceptance in groups of typical developing pupils. Carter et al. (2014) reported a high level of satisfaction with special solutions among both parents and teachers, as well as regular classroom placement for children with ASD. Facilitatory factors were found to be related to the learning practice and skills of the teachers, while obstacles were most often linked to the child’s characteristics. Burgstahler and Russo-Gleicher (2015) argued that both legislation and social policy advocating for inclusion have contributed to increased admission of pupils with disability, including pupils with ASD in schools. However, literature regarding how the needs of students with ASD can be met in classes is currently scarce.

In one study, Haimour and Obaidat (2013) found that teachers had an acceptable to weak knowledge about ASD. A study by Corkum et al. (2014) indicated that teachers have difficulty meeting the varying needs of children with ASD within a framework of inclusive education. In addition, the researchers found that teachers emphasized the need for versatile professional development to be available when needs arise. Sanahuja-Gavaldà et al. (2016) reported that barriers to inclusion often depend on how the school organizes support and teacher cooperation during this process. Schlieder et al. (2014) reported that the large increase in the number of children with ASD affects inclusion in mainstream schools, and that there is a need for teachers who are skilled in evidence-based methods to support pupils with ASD. The researchers proposed that an interactive website promoting social skills could contribute to increased involvement in school and reduce isolation and bullying among young people with ASD (Schlieder et al. 2014).

The European Agency (2018) proposed that opportunities for peer interaction can deepen friendships and a sense of belonging between pupils with and without ASD in inclusive environments. De Boer and Pijls (2016) reported significant differences in regular classrooms between pupils with ADHD and ASD and typically developing peers in peer acceptance, with ADHD and ASD pupils scoring higher on peer rejection. Typically developing pupils expressed neutral attitudes towards peers with ADHD or ASD. The results also indicated that pupils’ rejection and attitudes towards peers were significantly related to each other (De Boer and Pijls 2016). Increased awareness of autism among peers has been proposed as a strategy to prevent pupils with ASD being isolated and bullied. Dillenburger et al. (2017) examined 3353 11-year-old to 16-year-old pupils and found that awareness of autism was higher among teenagers (80%) than younger children (50%). Many children knew someone with autism (50%) and generally indicated positive and supportive attitudes. Peers believed bullying was a problem but wanted to help those who were bullied (Dillenburger et al. 2017).

### School Absence for Pupils with ASD

Munkhaugen et al. (2017) reported that refusal to attend school among pupils with ASD has only been studied to a limited extent, but is considered a serious problem. The results revealed that school refusal was significantly higher among pupils with ASD compared with typically developing pupils (Munkhaugen et al. 2017).

Several previous reports have indicated that school absence for pupils with ASD in Sweden has increased, and continues to increase (National Agency for Education 2013; Swedish Schools Inspectorate 2012; The Autism and Asperger Association 2016). Hebron and Humphrey (2014) reported that people with ASD have an increased risk of developing mental health difficulties, but the research is currently inadequate. In addition, the researchers reported that adolescents with ASD experienced significantly higher rates of depression, anxiety and anger than a group of adolescents with dyslexia (Hebron and Humphrey 2014). The group with ASD also exhibited lower self-concept scores than a group without special education needs (Hebron and Humphrey 2014). Mental health can correlate with factors such as social relationships and a lack of routines. Coping strategies could also help pupils overcome the difficulties they face. The researchers concluded that these factors contribute to school absenteeism for pupils with ASD (Hebron and Humphrey 2014). Sunera et al. (2012) reported that somatic problems and anxiety were the most common causes of school absenteeism, as well as conflict with peers and bullying. Compounding effects were found between gender and diagnosis. These findings indicated the importance of early identification and knowledge about the pupil’s school day (Sunera et al. 2012).

Hampshire et al. (2011) reported that deficits in organization and problem solving skills at school may lead to frustration and problem behaviors. Thus, increased barriers at school can lead to absence from school.

### Challenges, Demands and Obstacles in Education

A substantial amount of research on ASD and social skills has focused on school-aged males, whereas there have been few published studies on female teenagers with ASD. Females with ASD risk developing symptoms and experiencing major challenges in socialization and communication as social demands become increasingly complex over time. Jamison and Shuttlers (2017) reported significant improvements in social skills, self-perception and quality of life as children with ASD develop. The transition to high school is a common cause of stress and anxiety, which can be intensified for pupils with ASD. Peters and Brooks (2016) focused on experiences of the transition to secondary school from the parents’ perspective, and reported that factors such as anxiety, bullying, friendship and school support influenced pupils’ transition to secondary school. Girls with ASD exhibited unique problems that were typically not seen among boys. This may indicate that girls with ASD need special attention from educational and health services. Pupils with ASD and other types of developmental delay are at an increased risk of a number of challenges, such as motor, sensory and social functions, which can affect their ability to work in school. Anxiety and support strategies were two themes highlighted by five boys and one girl in Foulder-Hughes and Prior’s study (2014). Although pupils were able to make suggestions for strategies, their concerns outweighed the proposals (Foulder-Hughes and Prior 2014). In addition, the results revealed that sport education at secondary school was something that worried pupils in transition from primary to secondary school (Foulder-Hughes and Prior 2014).

Sedgewick et al. (2016) investigated two groups in special classes. Girls with ASD exhibited similar levels of social motivation and friendship as girls without ASD. In contrast, boys with ASD reported that they were less motivated to engage in social contact and had qualitatively different friendships compared with boys without ASD and girls with and without ASD. However, girls with ASD reported high levels of aggression within their friendships, indicating that girls with ASD may struggle to identify and manage conflict in social relationships (Sedgewick et al. 2016).

A study by Ranson and Byrne (2014) evaluated an anti-stigma program that focused on knowledge, attitudes and behaviors of teenagers with high-performing autism in primary school classes. The results indicated that the program had a positive impact on knowledge and attitudes, but had less impact on behavioral intentions toward other girls with high functioning autism. The researchers concluded that the anti-stigma program could be useful for supporting girls with high-functioning autism.

A study by Holcombe and Plunkett’s (2016) indicated that many teachers lack an understanding of how to identify individual needs and appropriate supportive strategies, despite pedagogical experience and extensive knowledge of ASD. The researchers pointed out the importance of common understanding of pupils’ strengths, identifying challenges, supportive strategies and specific goals for achieving success. Humphrey and Symes (2010) reported that pupils with ASD face a number of obstacles that can prevent them from getting the most out of their education. In particular, previous research has suggested that pupils with ASD are more likely to be bullied and receive less consistent social support than children with other or no special educational needs (Dillenburger et al. 2017). The perception of support for pupils, their relationships and lack of trust in others are considered to be important issues for staff to consider (Holcombe and Plunkett 2016).

O’Hagan and Hebron (2017) proposed that schools need to be aware of the social experiences of pupils with ASD, and how they can be integrated throughout the school environment. Saggers (2015) emphasized the importance of providing quiet areas with fewer people, and providing opportunities for pupils with ASD to work in smaller groups during the school day. Santos et al. (2016) reported that the social climate in the classroom is a key factor in the development of inclusive education considered. Many factors contribute to the classroom climate, but relational factors appear to exert the strongest influence on actions, norms and values, social interactions and learning processes. The results indicated that relationships between teachers and pupils with a need for educational support differ from those between teachers and typically developing children, but also differ from those of other groups of pupils in need of support (Santos et al. 2016). There were, however, no significant differences between mainstream teachers and special educators. Bryans-Bongey (2018) explored applications in teaching pupils who need concrete and visual methods for optimal and expressive communication. Teachers of students with ASD, as well as other students in need of visual support, can use various applications to support their teaching.

## Study Purpose and Research Questions

The aim of the current study was to explore parental perspectives of school absence in relation to goal fulfillment, challenges, demands and obstacles in education for children with ASD. Four questions were examined: (1) What correlations exist between school absence, grades and types of school? (2) What are the causes of school absence? (3) What are the relationships between pupils’ gender and the achievement of approved grades in English, Swedish and mathematics? (4) What are the relationships between pupils’ gender and educational support?

## Methods

### Participants and Procedu***re***

To examine schooling for pupils with ASD from parents’ perspectives and investigate our research aim and questions, Professor Mona Holmqvist, Malmö University, conducted an anonymous web-based questionnaire on behalf of the Swedish National Autism Association (2016). The Autism and Asperger Association in Sweden had approximately 16,000 members in 2016, including parents, people with a diagnosis of ASD, relatives of people with ASD, and professionals. The number of members increased to 18,000 in 2019. The questionnaires were sent to all members with an e-mail address, targeting parents with children aged 6–21 years attending primary or secondary school and diagnosed with ASD. Information about the total number of parents with children in this age group was not available. The web questionnaires were distributed between February 29 and March 13, 2016, to members of the Autism Association, to examine the schooling of their children. 2, 428 people answered questions about whether their child belonged to the target group or not. After this selection process, the target group consisted of 1799 individuals who answered the questionnaires, and this information constituted the data examined in the current study.

### Data Analysis

The questionnaire consisted of 19 questions. Of these, 15 questions were rated on an ordinal scale, and three questions were rated on a 5-point Likert scale (for example: “The support your child gets from school” with answer options 1 = very badly and 5 = very good). Three questions had an “other” option (for example: “What do you perceive that school absence is primarily based on?” and “In what way do you think support for your child could be improved?”, where the seventh option was “other”).

The questionnaire responses were analyzed using quantitative methods (Creswell 2013). Cross tabulation and chi-square analyses were performed using the IBM SPSS statistical software package (v. 24; IBM SPSS, Armonk, NY, USA) to analyze patterns in parents’ reported perspectives regarding their children’s schooling. The level of statistical significance was set at p < .05. To explore the first research question “What correlations exist between school absence, grades and the type of school?”, analyses of the following items were performed: “What kind of school does the child attend?”, “Has the child been absent from lessons for reasons other than illness or other granted leave?”, and “Has the child completed approved grades in Swedish, English and mathematics?”. To explore the second research question “What are the causes of school absence?” analysis of the following item was performed: “What do you think school absence is primarily due to?” (multiple answers possible). To explore the third research question, “What are the relationships between pupils’ gender and the achievement of approved grades in English, Swedish and mathematics?” analyses of the following items were performed: “Has the child completed all approved grades in Swedish, English and mathematics?”, and “What is the child’s gender?”. To explore the fourth question, “What are the relationships between pupils’ gender and educational support?” analyses of the following items were performed: “Which of the following does your child have access to as teaching support?” (multiple answers are possible); “In what way do you think the support could be improved?”, and “What is the child’s gender?”. The purpose of these analyses was to determine whether there were any significant differences between children at different school types regarding school absenteeism and grades. Furthermore, it was examined whether there were any gender-related differences in grades and school absenteeism, or any gender-related differences in educational support. Chi-square statistics (*X*^*2*^) were used for these analyses.

### Ethical Considerations

The National Autism and Asperger Association in Sweden owns the dataset examined in the current study, and Malmö University purchased access to the entire database and has subsequently received ethical approval from the Regional Ethics Board in Lund, Sweden (Dnr 2016/838). The questionnaires were distributed online by the marketing and community information consulting companies Demoskop to all members with e-mail address, targeting parents with children aged 6–21 years, and including information about the aim of the survey. All information used in this article has been treated confidentially in accordance with ethical rules in Sweden (Vetenskapsrådet 2018) and the guidelines of the Regional Ethics Board.

## Results

The results from the quantitative analysis are based on responses from 1799 members of the National Autism Association in Sweden. Four main research questions were examined: (1) What correlations exist between school absence, grades and school forms?; (2) What are the causes of school absence?; (3) What are the relationships between pupils’ gender and the achievement of approved grades in English, Swedish and mathematics?; and (4) What are the relationships between pupils’ gender and educational support?

### Background

The table below (Table 1) shows the rates of parents’ responses regarding the school type attended by their children. The majority of children (1318/75.3%) attended primary or secondary schools. Only 319 (18.2%) pupils attended primary or secondary schools for students with learning disabilities. The option, “other” was likely to include other special solutions, such as resource schools, individual teaching and adapted study. The internal dropout number was 28 respondents.

**Table 1:** Response rate for school form (N = 1751)

School forms	N (%)
Primary school	1126 (64.3%)
Primary school for learning disabilities	216 (12.3%)
Secondary school	192 (11.0%)
Secondary school for learning disabilities	103 (5.9%)
Other solutions	114 (6.5%)
Total	1751 (100%)

### School Absence, School Forms and Grades

The *first question* examined the correlations between school absence, grades and school forms. The data in Table 2 revealed a significant difference between school type and absence from school (p = .027) and a significant difference between absence from school and approved grades (p =  − .137).

**Table 2:** Correlation between school absence, school type and approved grades

Pearson’s correlation	School forms	Absence	Grade
School forms	1	.027**	− .009
Absence	.027**	1	− .137**
Grade	− .009	− .137**	1

**Correlation is significant at the 0.05 level (two-tailed)

### School Absence Without Illness and Solely with Illness

The *second question* analyzed the rates of school absence and the reasons underlying absence. Table 3 shows that school absence without illness in primary school (51.3%) and secondary school (57.6%) did not significantly differ. Pupils at secondary school exhibited higher rates of school absence for reasons other than illness compared with pupils in primary school. School absence due to illness was the main cause of absence in primary schools for students with learning disabilities (83.8%) but the rate of absenteeism for reasons other than illness increased when students attended secondary schools for students with learning disabilities (36.3%). Absence due to illness (47.0%) or reasons other than illness (53.0%) from other types of school solutions, such as resource schools, did not deviate significantly from the rates of absence in primary or secondary schools. The internal dropout number was 50 respondents.

**Table 3:** School absence for reasons other than illness and absence due to illness in relation to school types (N = 1749)

	Absence without illness	Absence solely with illness	Total
Primary school	577 (51.3%)	548 (48.7%)	1125 (100%)
Primary school for learning disabilities	35 (16.2%)	181 (83.8%)	216 (100%)
Secondary school	110 (57.6%)	81 (42.4%)	191 (100%)
Secondary school for learning disabilities	37 (36.3%)	65 (63.7%)	102 (100%)
Other solutions	61 (53.0%)	54 (47.0%)	115 (100%)
Total	820 (46.9%)	929 (53.1%)	1749 (100%)

The results shown in Table 4 revealed a gender difference, where girls with ASD exhibited higher rates of absence (54.6%) for reasons other than illness than boys with ASD (43.9%). The analysis revealed that girls exhibited significantly more short absences for reasons other than illness compared with boys (p =  − .096**). For continuous periods of absence longer than 4 weeks, there was no significant difference between boys and girls.

**Table 4:** School absence without illness among boys and girls (N = 1737)

	Absence without illness		
	Yes	No	Total
Boys	569 (43.9%)	725 (56.1%)	1294 (100%)
Girls	242 (54.6%)	201 (45.4%)	443 (100%)
Total	811 (46.6%)	926 (53.4%)	1737 (100%)

As parents were able to choose more than one response option, the response rate was N = 2338 (Table 5). Parents stated that the most common reason for children’s school absence was a lack of autism competence (26.6%), lack of adaptation of the school environment (24.3%) and support in learning situations (23.5%) and social situations (23.8%). Bullying was mentioned to a limited extent as a cause of absence (4.8%). The option “other” (14.0%) could indicate the need for extra rest, stress, fatigue, anxiety, depression, failing to meet challenges, sensory stress, or sleeping problems.

**Table 5:** Reasons for absence (N = 2338)

Reason for absence	N (%)
Lack of autism competence of staff	478 (26.6%)
Lack of adaptation of the school environment	477 (24.3%)
Lack of support in learning situations	437 (23.5%)
Lack of support in social situations	429 (23.8%)
Other	252 (14.0%)
Key person in school absent	229 (12.7%)
Bullying	86 (4.8%)

### School Types, Approved Grades in Subjects and Boys and Girls

The *third question* analyzed the relationship between pupils’ gender and the achievement of approved grades in Swedish, English and mathematics. The findings shown in Table 6 indicated that approximately 50% of pupils with ASD achieved approved grades in Swedish, English and mathematics. Primary and secondary schools for students with learning disabilities were the school types for which the probability of failing to reach the approved level was highest (p = .000). One potential explanation for this finding is that grades are not given in the same way in primary and secondary schools for learning disabilities compared with regular primary or secondary schools. First, not all grading steps are used, and grades tend to be given out on request. There are no grade statistics for students at primary and secondary schools for pupils with disabilities.

**Table 6:** School types and approved grades in Swedish, English and mathematics (N = 1605)

	Yes	No	Total
Primary school	557 (53.6%)	482 (46.4%)	1039 (100.0%)
Primary school for learning disabilities	70 (39.5%)	107 (60.5%)	177 (100.0%)
Secondary school	108 (56.8%)	82 (43.2)	190 (100.0%)
Secondary school for learning disabilities	33 (35.1%)	61 (64.9%)	94 (100.0%)
Other solutions	28 (26.7%)	77 (73.3%)	105 (100.0%)
Total	796 (49.6%)	809 (50.4%)	1605 (100.0%)

The results shown in Table 7 revealed no significant differences between boys and girls in the rates of achieving approved grades in Swedish language, English language and mathematics. However, a large proportion of students of both genders did not achieve approved grades in these three subjects.

**Table 7:** Boys, girls and approved grades in Swedish, English and Mathematics (N = 1597)

	Approved grades		Total
	Yes	No	
Boys	584 (49.5%)	596 (50.5%)	1180 (100.0%)
Girls	207 (49.6%)	210 (50.4%)	417 (100.0%)
Total	791 (49.6%)	806 (50.4%)	1597 (100.0%)

The *fourth question* analyzed the relationship between pupils’ gender and educational support. Parents reported the following types of educational support: adapted study programs, adapted pedagogy, and support from special needs teachers/special educators, adapted teaching materials, resource person, special teaching groups, resource schools, individual teaching and home teaching. Table 8 shows the distribution between different forms of educational support. In particular, parents reported that pupils with ASD received support from teachers adapting pedagogy in the classroom (54.4%), support from special needs teachers and special educators (42.8%), and support from adapted teaching materials (38.7%) and resource persons (37.6%). Several response options were available.

**Table 8:** Access to different forms of educational support in teaching (N = 1799)

Educational support	N	Percent (%)
Adapted study program	535	29.7
Adapted pedagogy	978	54.4
Support from special needs teacher or special educator	770	42.8
Adapted teaching materials	697	38.7
Resource person	676	37.6
Special teaching group		29.1
Resource school	202	11.2
Individual teaching or home teaching	185	10.3

The detailed analyses revealed that it was more common for boys (80.6%) than girls (19.4%) to be assigned to a resource person, and boys (81.6%) were more often given individual or home teaching compared with girls (18.4%). The difference between boys and girls was greatest for adapting classroom teaching (74.4% and 25.6% for boys and girls, respectively). Regardless of the type of educational support, boys were reported to receive educational support more often. Among the types of educational support directed to boys and girls, the most common forms of support were adapted pedagogy, special needs teacher / special educator and resource person.

The questionnaire also contained a question about how parents assessed their children’s wellbeing at school, and a question about how they assessed the support provided by the school. The analysis revealed a significant difference in wellbeing between boys and girls, such that boys (79.5%) were more likely to be reported to thrive at school compared with girls (21.5%). The difference was significant (p = .126**). The results also revealed a significant difference between boys and girls regarding the educational support given to pupils (p =  − .099**; boys: 78.6%; girls: 21.4%). However, the overall analysis of wellbeing and educational support revealed that, in the parents’ view, children were generally comfortable at school and that educational support was good, but students with ASD felt better at special schools for students with learning disabilities, compared with ordinary primary and secondary schools.

## Discussion

The current study aimed to explore parental perspectives regarding school absence, approved grades, challenges, demands and obstacles in education for their children with ASD. The sample consisted of 1,799 parents with children with diagnosed ASD between the ages of 6 and 21 years.

In the current study, most students with ASD attended standard primary or secondary schools. Some students received other forms of teaching, such as primary and secondary schools for pupils with learning disabilities (18.2%) and resource schools (6.5%). Thus, we were able to compare parents’ perceptions regarding the best form of schooling for children with ASD. Parents expressed the highest preference for special schools known as “resource schools”, which 65% of the parents believed was the optimal type of school (The Autism and Asperger Association 2013). Carter et al. (2014) reported that parents and teachers were typically satisfied with special solutions, as well as regular classroom placement. The factors facilitating the education of students with ASD are related to the learning practice and skills of the teachers. Göransson and Nilholm (2014) reported that the ways in which classes and teachers function are more important for students with ASD than the way in which schools are organized at a general level. Thus, there is a “gap” between parents’ perceptions, research and international conventions regarding the types of school at which students with disabilities can receive the most effective education. Waddington and Reed (2017) reported that typically developing pupils do not have greater academic success than pupils receiving education via special solutions. The current results indicated that primary and secondary schools for students with learning disabilities were the type of school with the highest probability that students would not achieve the approved grade level. However, a direct comparison with typical primary and secondary schools may be inappropriate, as the provision of grades at special schools differs from that at typical schools.

The current results indicate that the rate of school absenteeism among students with ASD in primary school is relatively high, and increases when pupils start secondary school. School attendance, on the other hand, was significantly higher for students attending primary or secondary schools for pupils with learning disabilities. In addition, the results revealed a difference in school attendance rates between girls and boys with ASD. Previous reports have indicated that absenteeism is increasing for students with ASD over time. The present study further indicates that absenteeism is continuing to increase. Hebron and Humphrey (2014), in accord with Sunera et al. (2012), reported an increased risk of developing mental health difficulties, somatic problems, anxiety and anger among pupils with ASD, and that these factors contributed to school absenteeism. Sunera et al. (2012) also reported that bullying caused school absence in some cases. In the present study, bullying was less prominent as a cause of school absenteeism. Early identification and knowledge about the pupil’s school day are important and different forms of obstacles during the school day can lead to frustration and problematic conditions, causing pupils to avoid going to school (Hampshire et al. 2011; Sunera et al. 2012). The results of the present study suggest that girls with ASD may be particularly vulnerable, potentially leading to school absenteeism, at least for short durations. Peters and Brooks (2016) reported that girls with ASD exhibited unique problems that are not typically seen among boys, indicating that the school situation and mental health needs of girls require special attention.

The results of the present study revealed that slightly more than 50% of students with ASD achieved approved grades in core subjects. Boys and girls did not differ in this regard, but it is striking that such a large proportion of pupils did not achieve approved grades. Jamison and Shuttlers (2017) reported that the transition to high school is a common cause of stress, which can contribute to missed grades. The current results indicate that knowledge about autism, adaptation of study situations and educational support is lacking. Holcombe and Plunkett (2016) reached a similar conclusion. Humphrey and Symes (2010) reported that pupils with ASD faced a number of obstacles preventing them from getting the most out of their education. This was also evident in the present research, including a lack of sufficient skills and educational support to meet the needs of the pupils. This situation could lead to higher absenteeism and less subject knowledge, resulting in a failure of students to achieve the approved grades. The parents in the current study emphasized that adapted pedagogy, such as clarifying schedules and oral exams, teaching aids or other equipment and support from special teachers were the forms of support that benefitted children the most. As educational support for boys and girls appeared to differ, it is particularly important to pay attention to the study situation for girls with ASD. LaBarbera (2017) suggested that special educators should seek a more in-depth understanding of parents’ perspectives so that school professionals can use the information proactively to create a meaningful partnership that meets the academic and social needs of pupils with ASD. Bloom (2012) proposed that school leadership has the potential to open doors for pupils with ASD by providing them with confidence that enables them to reach their full potential.

## Limitations and Future Research

The number of members in the National Autism and Asperger Association was 16,000 in 2016, and the number of parents who reported that they were currently caring for children was 2428 (i.e., 11% of all members). Of the 2428 parents with children, 1799 of parents with children in the study age group (6–21 years) answered the questionnaire. The difference between these two numbers may be due to some parents having children that were younger or older than the target age range. There may be other parents in the national association who have children in the current age group, but who did not respond to the questionnaire, and no information regarding the number of eligible parents who were not included is available. In addition, there was a source of error related to the grades in Swedish, English and mathematics, which could not be differentiated due to the design of the questionnaire, which only distinguished whether or not the pupils received approved grades in all three subjects. However, despite these limitations, the dataset was sufficiently large for analyses to be conducted and conclusions to be drawn. There is inevitably some uncertainty in the data, and several important questions remain: How well do these parents’ experiences match those of other parents? Would the results be different at another time and with other age groups? Which parents were most likely to answer the questionnaire? The results clearly indicated that parents’ perception of their children’s schooling was more negative that that reported in a survey conducted by Demoskop in 2014, and parents’ activity about school issues increase when the child starts school (The Autism and Asperger Association 2016). Since the data used for analysis were already collected, the content and design of the questionnaire could not be modified. In future questionnaire studies, it may be useful to ask additional questions, and to provide other response options. One advantage of utilizing the questionnaire from the National Autism and Asperger Association is that the same questionnaire has been repeated on several occasions, meaning that changes over time can be tracked.

Future research on schooling for pupils with ASD using quantitative methods should be conducted to further elucidate the situation for pupils in upper secondary school. Respondents could include pupils as well as different professional staff within the school. Another important research topic is investigating the perspectives of young people regarding educational, social and physical accessibility in school. It may also be valuable to examine girls’ schooling over time.

## Conclusions

The findings of the current study contribute new knowledge to current understanding about where students with ASD receive their teaching, as well as school absenteeism and the reasons pupils stay at home. The current results also highlight gender-related differences regarding absence, educational support and the level of comfort felt by pupils with ASD at school. The results suggested that student wellbeing was higher in schools for pupils with learning disabilities, possibly due to smaller student groups and greater teacher density, as well as a higher level of teacher competence regarding ASD. Importantly, the findings revealed that approximately 50% of pupils in primary and secondary school did not achieve approved grades in core subjects. This finding has major consequences for further studies. The current results also reinforce the notion that knowledge is currently lacking about autism and the ways in which teaching can be best organized to meet the needs of students with ASD. In the current study, it was clear that the school situation for pupils with ASD, particularly girls, requires further examination. Despite several negative features of schooling for pupils with ASD, parental reports indicated that schooling generally works well in terms of both wellbeing and educational support for most students.
